# HbA1c level cannot predict the treatment outcome of smear-positive non-multi-drug-resistant HIV-negative pulmonary tuberculosis inpatients

**DOI:** 10.1038/srep46488

**Published:** 2017-04-13

**Authors:** Ken Tashiro, Nobuyuki Horita, Kenjiro Nagai, Misako Ikeda, Masaharu Shinkai, Masaki Yamamoto, Takashi Sato, Yu Hara, Hideyuki Nagakura, Yuji Shibata, Hiroki Watanabe, Kentaro Nakashima, Ryota Ushio, Akimichi Nagashima, Atsuya Narita, Nobuaki Kobayashi, Makoto Kudo, Takeshi Kaneko

**Affiliations:** 1 Department of Pulmonology, Yokohama City University Graduate School of Medicine, Yokohama, Japan; 2 Respiratory Disease Center, Yokohama City University Medical Center, Yokohama, Japan

## Abstract

We conducted a single-center retrospective cohort study to evaluate whether the HbA1c level on admission could predict the in-hospital treatment outcome of smear-positive non-multi-drug-resistant HIV-negative culture-proven pulmonary tuberculosis inpatients. Our standard regimens under the direct observation were HRZE or HRE for the first two months followed by combination therapy with isoniazid and rifampicin. Our cohort consisted of consecutive 239 patients consisted of 147 men and 92 women with a median age of 73 years. The HbA1c level of patients whose HbA1c was above 7.0% on admission showed clear declining trends after admission. HbA1c on admission had no Spearman’s rank correlation with time to discharge alive (r = 0.17) and time to becoming non-infective (r = 0.17). By Kaplan-Meier curves and a log-rank trend test, HbA1c quartile subgroups showed no association with times to discharge alive (p = 0.431), becoming non-infective (p = 0.113), and in-hospital death (p = 0.427). Based on multi-variate Cox analysis, HbA1c on admission had no significant impact on time to discharge alive (hazard ratio = 1.03, 95% CI 0.89–1.20, p = 0.659), becoming non-infective (hazard ratio = 0.93, 95% CI 0.80–1.06, p = 0.277), and in-hospital death (hazard ratio = 0.68, 0.43–1.07, p = 0.097). In conclusion, the HbA1c level on admission did not seem to affect in-hospital tuberculosis treatment outcomes in Japanese cohort.

Tuberculosis (TB) is still a major concern for healthcare providers and patients. Even though the global TB incidence peaked in around 2003, approximately nine million persons develop TB every year^1^. Pulmonary tuberculosis is the most common form of TB infection, and causes symptoms such as persistent fever and chronic wet cough. The known risk factors for TB infection are HIV, malignancies, malnutrition, high age, and the use of immunosuppressants^2^,^3^,^4^. Besides these risk factors, diabetes mellitus (DM) is a considerable risk factor for tuberculosis, especially in developed countries^5^. Compared with people without diabetes, people who have diabetes are estimated to have an approximately three-fold risk of developing active TB^5^. In addition to increasing the risk for new TB infection, DM deteriorates the treatment outcomes of pulmonary tuberculosis^6^,^7^.

The currently recommended standard regimen for newly diagnosed pulmonary TB is eight weeks of isoniazid, rifampicin, pyrazinamide, and ethambutol (HRZE) followed by 18 weeks of isoniazid and rifampicin^2^,^8^. However, an additional eight weeks of isoniazid and rifampicin are recommended for patients with DM. This is because previous research has revealed that DM is a risk factor for treatment failure, recurrence, death, and persistent TB culture positive^7^. Controlling TB of patients who have concomitant DM is an important issue relevant to both patient care and public health. Currently, we have some clinically available examinations to check DM control^5^,^6^. Among them, the beta-N-1-deoxy fructosyl component of hemoglobin (HbA1c) is the most reliable and prevalent single indicator for DM control. This reflects hyperglycemia during the previous two or three months^9^,^10^. We hypothesized that the HbA1c level on admission can predict the in-hospital treatment outcome of smear-positive HIV-negative tuberculosis patients who are treated with the standard regimen. To evaluate this hypothesis, we conducted a single-center retrospective cohort study.

## Methods

This study was carried out in accordance with Ethics Review Procedures concerning Research with Human Subjects published in 2015 by Ministry of Health, Labour and Welfare, Japan. The Institutional Review Board of Yokohama City University approved the study protocol. The Ethics Review Procedures concerning Research with Human Subjects waived patient informed consent because of retrospective chart review design of this study.

### Inclusion criteria

In this study, we retrospectively obtained the data of consecutive smear-positive HIV-negative in-patients who were admitted to an isolation ward of our university hospital from January 2007 to December 2015 with a primary diagnosis of pulmonary tuberculosis. The primary cause of hospitalization was TB treatment and isolation, though some may have other acute co-morbidities. The tuberculosis was confirmed based on culture, though our observation began before we obtained the result of the culture. HIV status was routinely checked for every patient who was admitted to the ward. Exclusion criteria were as follows: 15 years old or younger, having already started anti-tuberculosis medication before admission, HIV positive, repeated admission due to tuberculosis, multi-drug-resistant tuberculosis, transferred out before the negative infectivity was confirmed, culture-negative TB, and treatment other than the two standard regimens described below^11^.

### Treatment

Our standard regimens under direct observation were HRZE or isoniazid, rifampicin, and ethambutol (HRE) for the first two months followed by combination therapy with isoniazid and rifampicin^8^. We usually selected the HRZE regimen, but the HRE regimen was used for patients at high risk of drug-induced liver injury based on the judgement of physicians^12^. The dosages of medications were as follows: isoniazid 5 mg/kg/day (maximum 300 mg/day), rifampicin 10 mg/kg/day (maximum 600 mg/day), pyrazinamide 25 mg/kg/day (maximum 1500 mg/day), and ethambutol 15–20 mg/kg/day (maximum 750 mg/day)^8^. These maximum dosage were recommended by The Japanese Society for Tuberculosis. All the regimens were provided as daily regimens.

All patients with high HbA1c level on admission and those who had diabetes on admission were treated following standard diabetes treatment guidelines including intensive insulin therapy, diet, metformin^9^,^10^. If necessary, we consulted diabetologists.

### Baseline assessment

We routinely conducted blood tests, sputum smear, and sputum culture on the day of admission. We routinely check HbA1c on admission for all patients who are admitted to our hospital due to TB. This is because local government requires us to report HbA1c level of all TB cases. The National Glycohemoglobin Standardization Program HbA1c was assessed since 2011. Until 2010, the Japanese Diabetes Society HbA1c was used in Japan instead. The Japanese Diabetes Society HbA1c was converted to the National Glycohemoglobin Standardization Program HbA1c by adding 0.4%^13^. A chest X-ray was also taken on admission for every patient. The existence of cavitation and infiltration bilaterality was judged by the physicians who took care of the patients independent from this study. Co-morbidity was determined by taking the history from the patients and family on admission or by referral letter on admission. Therefore, the value of HbA1c and the diagnosis of diabetes might be discordant with each other in our analysis.

The smear grade presenting bacterial load was classified into four classes based on the Japanese guidelines using the Ziehl-Neelsen stain (×1,000): (−), 0 acid-fast bacilli (AFB)/300 fields; (±), 1–2 AFB/300 fields; (+), 1–9 AFB/ 100 fields; (2+), 10–999 AFB/100 fields; and (3+), 10- AFB/field. Patients with smear grade (−) were regarded non-infective and were excluded from this study.

### Outcomes

Three main outcomes of our study were discharge alive, becoming non-infective, and in-hospital death. We collected sputum samples every week for the smear and the culture.

A patient under effective antibiotic treatment was discharged when the patient had at least three consecutive negative sputum AFB smears or three consecutive negative cultures taken on different days^14^.

The day of becoming non-infective was retrospectively determined as the day when we collected the first sample of the three consecutive negative samples.

HbA1c value was measured once a month.

### Statistics

The Spearmans’s rank correlation test was used to evaluate the correlations between HbA1c quartiles and other baseline characteristics.

We used the Wilcoxon signed-rank test to evaluate whether HbA1c decreased or not after admission.

The Spearman’s rank correlation coefficient (r) was used to check the correlation between HbA1c level on admission and time to discharge alive and between the HbA1c level and time to becoming non-infective. The r was interpreted as follows:|r| < 0.2, no correlation; 0.2 ≤ |r| < 0.4, weak correlation; 0.4 ≤ |r| < 0.6, moderate correlation; 0.6 ≤ |r| < 0.8, strong correlation; 0.8 ≤ |r|, excellent correlation.

We used the Kaplan-Meier curve and the log-rank trend test to compare time-dependent event outcomes, i.e., time to discharge alive, time to becoming non-infective, and time to all-cause death depending on HbA1c quartiles.

The Cox proportional hazard model was used to assess the impact of baseline characteristics and the treatment regimen on outcomes adjusted for co-variables. Parameters that had association with HbA1c quantile with a cutoff p value of 0.10 were used as co-variables in the model. Insulin use after admission was excluded because co-linearity with HbA1c was suggested. TB treatment regimen was included in the model regardless of the association with HbA1c quantile. The stepwise forward selection method with a cutoff p value of 0.20 was used. The sputum smear on admission was converted to the binary outcome, i.e. (±)/(+) or (++)/(+++).

All analyses were performed using GraphPad Prism version 6.0 (GraphPad Software, San Diego, CA, USA) and BellCurve for Excel (SSRI, Tokyo, Japan).

## Results

### Patients’ background characteristics

During the observation period, 239 consecutive patients satisfied the inclusion criteria. Our cohort consisted of 147 men (62%) and 92 women (38%) with a median age of 73 (interquartile range (IQR): 51–82) years (Table 1).

Among the 239 patients, 111 (46%) had one or more pulmonary cavities and 178 (74%) had bilateral infiltration on X-ray (Table 1). The most common co-morbidity was diabetes, from which 70 patients (29%) in our cohort suffered, followed by chronic cardiac disease and chronic renal disease. The median HbA1c level was 5.9% (IQR: 5.6–6.6) and the median blood glucose was 109 mg/dL (96–133). In this cohort, 193 patients (81%) were discharged alive and 206 patients (86%) became non-infective before their death (Table 1).

After dividing patients into HbA1c quartile groups, the patients in the higher HbA1c quartile had lower total protein level and lower albumin level. Notably, patients belonging to the third quartile was elder than those in other quartile subgroups. The treatment regimen was not affected by the HbA1c level. Thirty two patients out of 45 patients (51%) in the fourth HbA1c quartiles were treated with insulin after admission (Table 1).

### Trends of HbA1c

**Figure 1:**
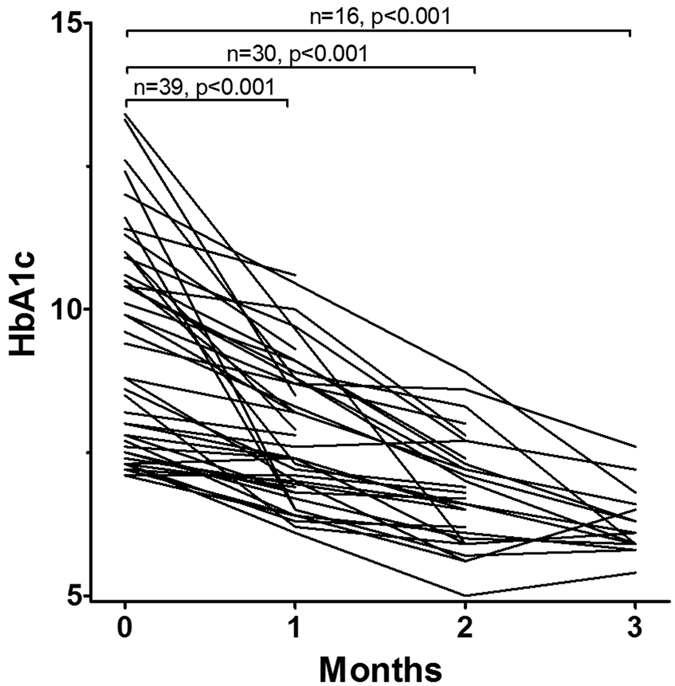
Change of HbA1c after admission. HbA1c levels of 44 patients whose HbA1c value on admission were above 7.0% and whose HbA1c were examined at least twice are presented. Wilcoxon signed-rank test.

Forty four patients had HbA1c on admission above 7.0%. HbA1c levels of all of these patients were measured twice or more during the first three months after admission. The trends of HbA1c of these 44 patients are presented in Fig. 1. The HbA1c level showed clear declining trends (p < 0.001 for HbA1c at 1, 2, and 3 months after admission compared to HbA1c on admission).

### Discharge alive

**Figure 2:**
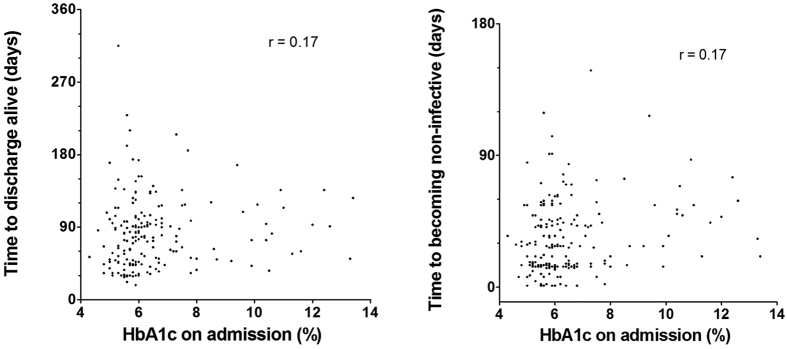
Correlations between HbA1c on admission, time to discharge alive and time to becoming non-infective. r: Pearson’s rank correlation coefficient.

**Figure 3:**
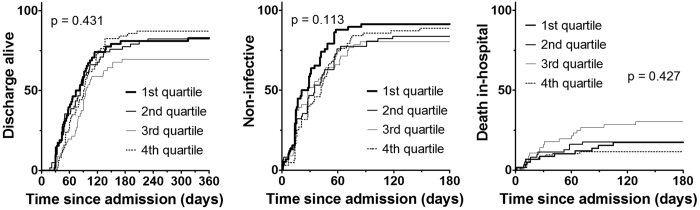
Kaplan Meier curves. p: log-rank trend test.

Eventually, 193 (81%) out of 239 patients were discharged alive (Table 1). HbA1c on admission had no meaningful correlation with time to discharge alive (r = 0.17, Fig. 2). Kaplan-Meier curves and the log-rank trend test did not reveal an association between HbA1c on admission and time to discharge alive (p = 0.431, Fig. 3). The hazard ratio (HR) estimated from a multi-variate Cox analysis was 1.03 (95% CI 0.89–1.20, p = 0.659) by 1% increase of HbA1c for time to discharge alive (Table 2).

### Time to becoming non-infective

Among 239 observed patients, 206 (86%) became non-infective (Table 1). The Spearman’s rank correlation test suggested no correlation between HbA1c on admission and time to becoming non-infective (r = 0.17, Fig. 2). The time to becoming non-infective was not significantly different in the patients belonging to the four HbA1c quartile subgroups based on the log-rank trend test (p = 0.113, Fig. 3). The HR for time to becoming non-infective by 1% increase of HbA1c was 0.93 (0.80–1.06, p = 0.277) (Table 2).

### In-hospital death

During the hospital course, 46 patients died. According to the death certificate, 39 patients died from TB. The other cause of death were two for liver cirrhosis and one for each of colon cancer, pneumonia, sepsis, brain infarction, myocardial infarction. In-hospital mortality was not associated with trend of HbA1c level (p = 0.631) (Table 1). Kaplan-Meier curves and a log-rank trend test did not reveal an association between HbA1c on admission and time to in-hospital death (p = 0.427, Fig. 3). However, the survival curve of the third quantile for in-hospital death seemed much worse than that of other three quantiles. The background characteristics of this subgroup featured by high age and low albumin level (Table 1). The HR estimated from multi-variate Cox analysis, which was adjusted for age, albumin and other co-variables, was 0.68 (95% CI 0.43–1.07, p = 0.097) by 1% increase of HbA1c for in-hospital death (Table 2).

## Discussion

Our pre-study hypothesis was that the high HbA1c level on admission can predict in-hospital TB treatment outcomes. This hypothesis is based on previous studies showing that co-morbid DM adversely affects the treatment outcomes of TB. The impact of DM on the treatment outcomes of TB has long been discussed. Baker *et al*. conducted a systematic review to summarize this topic in 2011^6^. According to this, data from 33 studies revealed that patients with DM have an adjusted risk ratio for death of 4.95 and a risk ratio for relapse of 3.89^6^.

However, the high HbA1c level on admission did not affect any in-hospital treatment outcomes in our analysis (Table 2, Figs 2 and 3). Therefore, we should discuss the gap between ours and previous studies^6^, which we mainly attributed to the following factors. First, our patients were in-hospital patients. Therefore, we observed relatively short-term outcomes only. Second, we performed tight glucose control using insulin if necessary^9^,^10^. Many patients had high HbA1c levels on admission. However, the HbA1c levels of all of these patients quickly decreased as shown in Fig. 2. Therefore, we suppose that the in-hospital therapeutic outcome of patients with high HbA1c value is not poorer than those without high HbA1c as long as the HbA1c is controlled properly. Similarly, diagnosis of diabetes also did not relate to three outcomes in our study (Table 2), despite the previous study suggested that DM is a considerable risk factor of poor outcomes^6^.

According to Kaplan-Meier curves, the survival curve of the third quantile for in-hospital death seems much worse than that of other three quantiles (Fig. 3). Patients in this third quantile were elder and had lower albumin level compared to patients in other groups (Table 1). High age and low albumin level were known risk factor of TB death^15^ After adjusting these factors, HbA1c was not related to outcomes (Table 2).

Our analysis suggested that serum albumin level had considerable impact on treatment outcomes of TB inpatients (Table 2). Our previous study showed that serum albumin is the strong predictive factor for survival^15^,^16^. In addition, the current analysis revealed that patients with high albumin level became non-infective faster than those with low albumin level (Table 2). Some other variables had marginally significant impact for outcomes in the Cox model (Table 2). For example, HRE regimen for discharged alive (P = 0.049), previous history of TB treatment for in-hospital death (P = 0.042), and chronic cardiac disease for in-hospital death (P = 0.037). However, given the large number of statistical tests for Cox model and possibility of alpha error, we should not overly emphasis these marginal significances.

The median duration of hospital stay was longer than 60 days in our cohorts. This may be almost twice as long as that reported in other studies^17^. This may because our cohort was mainly consisted of the elderly population and because as many as 46% of patients had a cavity lesion (Table 1). Our cohort may different from that in other countries, however, our cohort reflects TB epidemiology in Japan.

Limitations of our study should be mentioned. First, our analysis focused on HbA1c level on admission rather than diagnosis of DM. By definition, a high value of HbA1c does not completely equal to a diagnosis of DM. However, HbA1c is one of the DM diagnosis criteria and it is reasonable to assume that a large proportion of high HbA1c cases had DM. In addition, continuous HbA1c value more precisely reflected patient’s condition than binary DM diagnosis. Second, we assessed only in-hospital outcomes. Thus, long-term outcomes such as recurrence after end of treatment were not assessed. Third, tight glucose control using intensive insulin therapy is usually not feasible in an outpatient setting. Therefore, we should be cautious about applying the results from this study to an outpatient setting. Fourth, this study with moderate-scale retrospective cohort design was not conclusive and another large-scale prospective cohort study is warranted to validate our result. Fifth, our data was derived from a Japanese hospital, of which a large part of patients were elderly non-HIV population. Our cohort reflects Japanese epidemiology but does not reflect worldwide epidemiology^18^. Thus, it is not clear we can generalize the result to younger patients, persons in other areas, and HIV-infected patients. Sixth, although impact of latent TB among DM patients is another concern, our study could not directly contribute to this issue^19^.

In conclusion, we conducted this retrospective study with 239 smear-positive HIV-negative culture-proven tuberculosis inpatients and analyzed the impact of HbA1c on admission on the in-hospital outcomes. The in-hospital TB therapeutic outcome might not be poorer for patients with higher HbA1c level compared to those with lower HbA1c level as long as the glucose level is controlled properly.

## Additional Information

**How to cite this article:** Tashiro, K. *et al*. HbA1c level cannot predict the treatment outcome of smear-positive non-multi-drug-resistant HIV-negative pulmonary tuberculosis inpatients. *Sci. Rep.* **7**, 46488; doi: 10.1038/srep46488 (2017).

**Publisher's note:** Springer Nature remains neutral with regard to jurisdictional claims in published maps and institutional affiliations.

## Figures and Tables

**Table 1:** Patient background characteristics, treatment regimens, and key outcomes.

	All patients (N = 239)	HbA1c quartile				
		1^st^ (N = 58)	2^nd^ (N = 62)	3^rd^ (N = 56)	4^th^ (N = 63)	p
Age (years)	73 (51–82)	68 (36–81)	64 (42–81)	81 (74–84)	70 (58–80)	0.015
Sex (female)	92 (38%)	24 (41%)	23 (37%)	25 (45%)	20 (32%)	0.427
Cavity on X-ray	111 (46%)	27 (47%)	28 (45%)	21 (38%)	35 (56%)	0.461
Bilateral infiltration on X-ray	178 (74%)	38 (66%)	53 (85%)	43 (77%)	44 (70%)	0.926
Smear on admission (0.5, 1, 2, or 3)	2 (1–3)	2 (1–3)	2 (1–3)	2 (1–3)	2 (1–3)	0.154
Smear on admission ≥2	158 (66%)	37 (64%)	40 (65%)	36 (64%)	45 (71%)	0.395
Previous history of TB treatment	26 (11%)	10 (17%)	5 (8%)	9 (16%)	2 (3%)	0.052
Extra-pulmonary pulmonary TB	29 (12%)	6 (10%)	6 (10%)	7 (13%)	10 (16%)	0.296
Diabetes	70 (29%)	5 (9%)	7 (11%)	8 (14%)	50 (79%)	<0.001
Insulin before admission	9 (4%)	0 (0%)	0 (0%)	0 (0%)	9 (14%)	<0.001
Immunosuppression	27 (11%)	3 (5%)	10 (16%)	7 (13%)	7 (11%)	0.467
Chronic cardiac disease	41 (17%)	7 (12%)	6 (10%)	15 (27%)	13 (21%)	0.053
Chronic pulmonary disease	29 (12%)	11 (19%)	5 (8%)	9 (16%)	4 (6%)	0.110
Chronic liver disease	23 (10%)	7 (12%)	6 (10%)	4 (7%)	6 (10%)	0.568
Chronic renal disease	25 (10%)	7 (12%)	6 (10%)	3 (5%)	9 (14%)	0.848
Active malignancy	23 (10%)	8 (14%)	5 (8%)	4 (7%)	6 (10%)	0.443
Total protein (g/dL)	6.6 (5.9–7.2)	6.8 (6.1–7.4)	6.8 (6.1–7.4)	6.4 (5.4–7.1)	6.5 (5.7–7.2)	0.041
Albumin (g/dL)	2.8 (2.2–3.5)	3.15 (2.3–3.8)	3 (2.3–3.8)	2.5 (2.0–3.1)	2.7 (2.1–3.4)	0.005
Hemoglobin (g/dL)	11 (9.6–12.6)	10.6 (9.4–12.6)	11.4 (9.7–13.1)	10.8 (9.4–11.9)	11.1 (10.5–12.9)	0.109
Aspartate aminotransferase (IU/dL)	25 (19–43)	24 (18–39)	24 (19–36)	25 (22–35)	29 (18–49)	0.151
Creatinine (mg/dL)	0.66 (0.52–0.93)	0.65 (0.47–0.96)	0.62 (0.47–0.71)	0.74 (0.54–0.93)	0.71 (0.56–0.96)	0.075
HbA1c (%)	5.9 (5.6–6.6)	5.3 (5–5.5)	5.8 (5.7–5.9)	6.3 (6.1–6.4)	7.7 (7.1–9.9)	<0.001
Glucose (mg/dL)	109 (96–133)	101 (93–115)	108 (92–126.75)	107 (97–120)	149 (112–221)	<0.001
Treatment regimen						0.944
HRZE	155 (65%)	39 (67%)	41 (66%)	30 (54%)	45 (71%)	
HRE	84 (35%)	19 (33%)	21 (34%)	26 (46%)	18 (29%)	
Insulin after admission	32 (13%)	0 (0%)	0 (0%)	0 (0%)	32 (51%)	<0.001
Outcomes						
Duration of hospital course (day)	65 (IQR:39–95)	60 (IQR:38–91)	58 (IQR:31–91)	73 (IQR:34–94)	74 (IQR:49–105)	0.107
Discharged alive	193 (81%)	48 (83%)	51 (82%)	39 (70%)	55 (87%)	0.899
Died in-hospital	46 (19%)	10 (17%)	11 (18%)	17 (30%)	8 (13%)	0.899
Becoming non-infective	206 (86%)	53 (91%)	52 (84%)	45 (80%)	56 (89%)	0.631
Died before becoming non-infective	33 (14%)	5 (9%)	10 (16%)	11 (20%)	7 (11%)	0.631

HRZE: isoniazid, rifampicin, pyrazinamide, and ethambutol. HRE: isoniazid, rifampicin, and ethambutol. Median and interquartile range are presented for continuous variables. Numbers of patients and percentages were presented for binary variables.

**Table 2:** Cox proportional hazard analysis.

	Discharged alive	Becoming non-infective	In-hospital death
	HR (95% CI), p	HR (95% CI), p	HR (95% CI), p
Age (10 years)	0.98 (0.90–1.07), 0.616	1.03 (0.95–1.12), 0.522	1.44 (1.06–1.97), 0.021
Previous history of TB treatment	1.18 (0.72–1.95), 0.506	0.90 (0.56–1.45), 0.670	2.50 (1.03–6.03), 0.042
Diabetes	0.87 (0.58–1.29), 0.479	1.04 (0.70–1.55), 0.853	1.25 (0.44–3.61), 0.675
Chronic cardiac disease	0.90 (0.57–1.44), 0.668	1.16 (0.77–1.75), 0.485	2.06 (1.04–4.09), 0.037
Insulin before admission	1.23 (0.52–2.88), 0.637	1.10 (0.48–2.54), 0.815	1.14 (0.12–11.31), 0.909
Total protein (1 g/dL)	1.04 (0.84–1.29), 0.690	0.96 (0.80–1.16), 0.688	0.75 (0.50–1.12), 0.160
Albumin (1 g/dL)	2.32 (1.79–3.02), <0.001	1.88 (1.47–2.39), <0.001	0.13 (0.06–0.26), <0.001
Creatinine (1 mg/dL)	1.08 (0.96–1.21), 0.205	0.99 (0.87–1.11), 0.835	1.12 (0.90–1.04), 0.310
HbA1c (1%)	1.03 (0.89–1.20), 0.659	0.93 (0.80–1.06), 0.277	0.68 (0.43–1.07), 0.097
Blood glucose (10 mg/dL)	0.99 (0.95–1.03), 0.598	1.02 (0.98–1.05), 0.337	0.99 (0.93–1.06), 0.779
HRE regimen	0.67 (0.45–1.00), 0.049	1.03 (0.71–1.50), 0.867	0.81 (0.37–1.77), 0.596

HR: hazard ratio. HRE regimen: isoniazid, rifampicin, and ethambutol regimen was compared using isoniazid, rifampicin, pyrazinamide, and ethambutol as a reference.
